# Mitochondrial replacement techniques and Mexico's rule of law: on the legality of the first maternal spindle transfer case

**DOI:** 10.1093/jlb/lsw065

**Published:** 2017-03-23

**Authors:** César Palacios-González, María de Jesús Medina-Arellano

**Affiliations:** 1 Centre of Medical Law and Ethics, King's College London, Somerset House, Strand, London WC2R 2LS, UK; 2 Instituto de Investigaciones Jurídicas, Universidad Autónoma Nacional de México, Mexico City C. P. 04510, Mexico

**Keywords:** mitochondrial replacement techniques, mitochondrial replacement therapy, mitochondrial donation, maternal spindle transfer, pronuclear transfer, tri-parenthood, three parent babies, three parent IVF

## Abstract

News about the first baby born after a mitochondrial replacement technique (MRT; specifically maternal spindle transfer) broke on September 27, 2016 and, in a matter of hours, went global. Of special interest was the fact that the mitochondrial replacement procedure happened in Mexico. One of the scientists behind this world first was quoted as having said that he and his team went to Mexico to carry out the procedure because, in Mexico, there are no rules. In this paper, we explore Mexico's rule of law in relation to mitochondrial replacement techniques and show that, in fact, certain instances of MRTs are prohibited at the federal level and others are prohibited at the state level. *According to our interpretation of the law*, the scientists behind this first successful MRT procedure broke federal regulations regarding assisted fertilization research.

## Introduction

News of the first baby born after a mitochondrial replacement technique (MRT; specifically maternal spindle transfer [MST]) broke on September 27, 2016 and, in a matter of hours, went global.^1^ It was reported that the baby boy had been born to a Jordanian couple on the April 6, 2016 and was doing well. This biotechnological feat—the first of its kind in the world—was achieved by a team of scientists led by Dr. John Zhang. Zhang is the founder/CEO and medical director of the New Hope Fertility Clinic in New York City, NY. The Jordanian couple had resorted to Zhang after two of their children had died from Leigh's syndrome^2^—one at 6 years old and the other at 8 months old. At the present moment, the technical information we know about this case does not come from an academic paper; it comes from Zhang's short conference abstract and presentation, the statements that he and his team have given to the media, and the media reports from a late-breaking session at the American Society for Reproductive Medicine 2016 Scientific Congress.^3^ One of the things we know, and that is relevant for this paper, is that five oocytes were subject to MST and later fertilized via intracytoplasmic sperm injection. One embryo did not develop to the blastocyst stage, whereas four embryos did develop to the blastocyst stage. Of these four embryos, three were aneuploid and one was euploid. Euploidy is the presence of a normal complement of chromosomes. Aneuploidy is the presence of an abnormal number of chromosomes.

MRTs are only explicitly legal in the UK.^4^ Last year, after a long period of public consultation, both houses of parliament approved regulations put forward by the Department of Health, and these came into force on October 29, 2015.^5^ In order for MRTs to be carried out in the UK lawfully, clinics or centers must apply for and be granted a license, for each proposed procedure, from the Human Fertilisation and Embryology Authority. In the USA, this topic has been debated by a National Academy of Sciences panel, which recently published its recommendations in the *Ethical and Social Policy Considerations of Novel Techniques for Prevention of Maternal Transmission of Mitochondrial DNA Diseases*.^6^ Although the panel asserted that MRTs are, *in principle*, ethically acceptable, within certain limitations, Congress, through a federal spending bill, has effectively blocked them by prohibiting the FDA from considering applications to carry out these techniques:

None of the funds made available by this Act may be used to notify a sponsor or otherwise acknowledge receipt of a submission for an exemption for investigational use of a drug or biological product under section 505(i) of the Federal Food, Drug, and Cosmetic Act (21 U.S.C. 355(i)) or section 351(a)(3) of the Public Health Service Act (42 U.S.C. 262(a)(3)) in research in which a human embryo is intentionally created or modified to include a heritable genetic modification. Any such submission shall be deemed to have not been received by the Secretary, and the exemption may not go into effect. ^7^

The former, of course, affects MRTs in the creation of female embryos, as mitochondria are only maternally transmitted (as will become clear next). On the other hand, the wording of the Bill does not rule out the creation of male embryos, who do not transmit mitochondria to further generations. Even if this is so, a spokesperson of the FDA has said that ‘human subject research utilizing genetic modification of embryos for the prevention of transmission of mitochondrial disease cannot be performed in the United States in FY [fiscal year] 2016’.^8^ Thus, MRTs have effectively hit a road block in the USA.^9^ Additionally, it is important to bear two things in mind. First, no US federal legislation explicitly addresses human genetic modification. Second, in the USA, the Dickey-Wicker Amendment prohibits the use of *federal funds* for the creation of human embryos for research, or for research that results in the destruction of human embryos:

1. the creation of a human embryo or embryos for research purposes; or
2. research in which a human embryo or embryos are destroyed, discarded, or knowingly subjected to risk of injury or death greater than that allowed for research on fetuses in utero under 45 C.F.R. 46.208(a)(2) and 42 U.S.C. 289g(b).^10^

It seems that all of these regulatory issues, in addition to the FDA’s actions when a ‘similar’ procedure (cytoplasmic transfer^11^) was conducted in the USA in the recent past, prompted Zhang to carry out the procedure in Mexico.^12^ Why Mexico? Because the research and clinical practice of assisted reproduction in Mexico is very loosely regulated, if at all, and because Zhang's fertility clinic has two branches there: one in the State of Mexico City and the other in the city of Guadalajara, within the state of Jalisco. The procedure was carried out in the Guadalajara branch. Unfortunately, Zhang was quoted in the publication that broke the news as stating that he and his team went to Mexico because, in Mexico, ‘there are no rules’.^13^ Zhang was most likely referring to the fact that, in Mexico, MRTs are neither *specifically regulated* nor *specifically forbidden*; but even if this was his intending meaning, the media has since repeated *ad nauseam* that, in Mexico, there are no laws governing MRTs and assisted reproduction.

Against this background, in this paper we set out to examine MRTs in the context of Mexico's rule of law. Specifically, we explore whether Mexican law *prohibits* either of the two MRTs: MST and pronuclear transfer (PNT). This research is important in terms of regulatory background given that the first successful instance of MST occurred in Mexico and it is feasible that Zhang's team—or another team—will consider carrying out either this procedure or PNT, in Mexico again.^14^

In the first section of the paper, we present a brief account of mitochondrial DNA diseases and MRTs (if you are familiar with these topics then you can skip ahead to the second section). We explain how these techniques are carried out and describe two important characteristics of them. In the second section, we explore Mexico's rule of law in regard to MRTs. As stated above, we descriptively examine the legal status of MST and PNT.^15^ First, we very briefly describe Mexico's political composition and examine whether Mexico's highest national law, the Political Constitution of the United Mexican States (hereafter the Federal Constitution), specifically protects life from the moment of conception or fertilization.^16^ Secondly, we investigate the legality of MRTs from the perspective of federal laws. Thirdly, we identify those states in which local laws protect human life from the point of conception and fertilization, and investigate how MRTs interact with such local laws. We pay particular attention to the state of Jalisco, given that it was there that the MST was carried out. Fourthly, we examine MRTs in terms of Mexican laws regulating both human genome modification and human genetic engineering.

In the third section of the paper, the conclusion, we briefly present our main points and explore how Zhang's team's actions have affected the assisted reproduction debate in Mexico. Specifically, we discuss how their actions have helped those who wish to pass federal legislation to prohibit MRTs, and that would restrict access to assisted reproduction.

Before moving to the next section, we clarify that this paper does not engage with the ethics of MRTs^17^ or how they should be regulated on an international level.^18^ Although both authors find that carrying out MRTs is, *in principle*, ethically permissible—*one* of the authors has written on the ethical permissibility of MRTs and the other on the importance of embryo and stem cell research for unmet health needs—the focus of this paper is on MRTs and Mexico's rule of law.

## Mitochondria^19^

All eukaryotic organisms, humans included, possess a double membrane-bound organelle called mitochondria, which resides in the cytoplasm. This organelle, among other things, produces the energy cells need to work properly. Two important characteristics of mitochondria are that they are *solely* maternally inherited^20^ and that they possess their own DNA, which, again, is located outside the cell's nucleus. Whereas nuclear DNA accounts for 99.9 per cent of total human DNA, mitochondrial DNA (henceforth mtDNA), with its 37 genes, accounts for the other 0.1 per cent.^21^

Given that mitochondria are responsible for generating the energy cells need to work properly, it is not surprising that when they do not work as they should there may be adverse effects on human health, ranging from mild to devastating in their severity. Mitochondrial diseases are caused by the inadequate function of mitochondria. These diseases can occur because of problems within the mtDNA, itself, or because of the effect of the nuclear DNA on mitochondrial function.^22^ In this paper, we will only focus on problems related to the function of mtDNA, itself.

Roughly speaking, mitochondrial DNA diseases (henceforth mtDNA diseases) occur when enough mitochondria with *deleterious DNA mutations* exist for the production of energy to be insufficient for cells to work properly. Let's remember that each cell has many mitochondria. Deleterious mutations can (a) occur spontaneously during mtDNA replication, (b) be inherited, or (c) both. These mutations can occur across all mitochondrial genomes—a condition referred to as ‘homoplasmy’; or they can only occur in some mitochondrial genomes—a condition referred to as ‘heteroplasmy’.

Women with homoplasmic deleterious mutations will *always* pass this condition to their children, and those with heteroplasmic deleterious mutations will pass a *mix* of unhealthy and healthy mitochondria to their children. The severity of the mtDNA disease and whether it will manifest at all, in children of heteroplasmic women, depends on the type of mtDNA mutation and the load of deleteriously mutated mitochondria. Prediction on this outcome is difficult, since mitochondria are not inherited in a Mendelian manner; rather, their inheritance depends on a phenomenon called the ‘mitochondrial DNA genetic bottleneck^23^’.

It must be clear that, up to this point, we have discussed *mtDNA diseases*, in plural, because such diseases cannot be categorized in a singular fashion. Deleterious mutations in mtDNA can cause Leigh's syndrome, deafness, blindness, stroke, dementia, major organ failure, heart failure, and Leber's hereditary optic neuropathy, among other conditions.^24^ The UK Department of Health asserts that 1 in every 6500 children born in the UK has an mtDNA disease.^25^

As there is presently no cure for mtDNA diseases, women who have them (and are aware that they have them) and want to have *genetically related kin* face a difficult reproductive choice. Homoplasmic women know that if they reproduce naturally, all of their children will inherit their homoplasmic condition and will probably end up with a clinical manifestation of the disease. Heteroplasmic women, on the other hand, know that it is probable that their children will possess a deleterious mutant load that could cause the disease to manifest. Until now, women with mtDNA diseases who want to have *genetically related children* have had to choose between natural procreation or, where available, preimplantation genetic diagnosis (PGD)—with the latter option only available to heteroplasmic women.

The idea behind using PGD is that physicians are able to select an embryo that possesses no mutant load, or one that possesses a mutant load so small that the clinical manifestation of the disease will not occur.^26^ A problem with this approach is that it does not work for homoplasmic women, as implied above, and that, in the case of heteroplasmic women, it only works when there is enough data on the specific mtDNA mutation. This suggests that PGD is not useful for mutations that are uncommon or private. It must be obvious that there are other reproductive options for women affected by mtDNA diseases: egg and embryo donation. However, these forgo the maternal genetic link that is of paramount importance to some.

Another option (which was merely theoretical until recently) is for women to opt for one of two recently developed techniques that would allow them, if successful, to have children free from their mtDNA disease. These techniques are maternal spindle transfer and pronuclear transfer, and these have been jointly called ‘mitochondrial replacement techniques’.^27^ It is important to highlight that these techniques *only tackle mtDNA diseases*.^28^

In PNT, assisted reproductive techniques (ARTs) are employed to create two zygotes. One is created with the intending parents’ gametes (or the intending mother's egg and a donor's sperm). The second is created with an egg that has been donated from a woman^29^*without* an mtDNA disease, and the intending father's (or donor's) sperm. After fertilization occurs, the nuclear material of the egg and sperm are enclosed in different membranes, called the male and female pronuclei. These pronuclei are removed from both zygotes during the first 24 hours and prior to their fusion. The pronuclei that include the donor's nuclear material and the enucleated cell that was originally produced with the intending mother's oocyte are discarded. The intending parents’ pronuclei are then transferred to the enucleated zygote that was produced with the donor's egg. At this point, the intending parents’ nuclear material is housed in a cell possessing healthy mitochondria and is transferred into the intending mother or a surrogate.^30^

In MST, oocytes are obtained from the intending mother and a healthy donor through ARTs. The chromosomes of both oocytes, which at the moment of cellular division (metaphase II) are found to one side of the oocyte in a spindle shape group, are removed. The donor's chromosomes and the intending mother's enucleated oocyte are discarded. Afterwards, the intending mother's chromosomes are transferred to the donor's enucleated oocyte. The reconstructed egg, with healthy mitochondria, is then fertilized in vitro and transferred to the intending mother or a surrogate.^31^

Children born after PNT or MST will not have an mtDNA disease *if* during the procedures there was not enough carryover of deleteriously mutated mitochondria for the disease to clinically manifest.^32^ In this case, the donor's healthy mitochondria will be passed to future generations when women born after MST or PNT reproduce using their own eggs.

## Two relevant characteristics

Two characteristics of MRTs are relevant in terms of law and regulation. The first is that both PNT and MST can be instances of either germline or somatic modifications. As stated above, PNT and MST affect the germline, in the sense that they are modifications that *will be* inherited by future generations if a female individual produced through them reproduces. However, both techniques are somatic modifications when a male embryo is selected for, in the sense that the modifications *will not be* inherited by future generations if the individual produced through them reproduces.

It is possible to both select the sex of the embryo that will be created and select the sex of the embryo that will be transferred to the mother or a surrogate. Through PGD one can choose the sex of the embryo that will be transferred. This means that if post-MST a female embryo and a male embryo are produced and we want to avoid modifications being passed down, we can identify the male embryo and transfer it. On the other hand, through sperm sorting we can choose a sperm with an ‘X’ or a ‘Y’ sex chromosome and thus select the sex of the embryo that will be created. This technique works prefertilization and also allows us, for example, to create only male embryos.

The second characteristic that is relevant for law and regulation is the difference between PNT and MST in terms of the time at which they occur. Whereas MST involves oocytes, PNT involves zygotes. In MST, the donor's oocyte is destroyed for the sake of the intending mother's oocyte. In PNT, however, an early embryo is destroyed for the sake of another early embryo. In moral terms, this difference is of paramount relevance for those who hold that human embryos possess the same moral status as human persons. This difference between PNT and MST is also important because, as we will see below, certain jurisdictions protect ‘human life’ from the moment of fertilization.

## Mexico

The United Mexican States, commonly known as Mexico, is a federal republic of 32 states.^33^ The states’ powers, at both the local and the federal level, are divided into executive, legislative, and judicial powers. At the federal level, the executive power falls to the President; the legislative power falls to the Congress of the Union (henceforth Congress), which is divided into two chambers: the Senate and the Chamber of Deputies; and the judicial power falls to the Supreme Court of Justice (henceforth the Supreme Court).

Mexico's highest law is the Federal Constitution and in it (Articles 39 to 41) the form of government and the integration of the Federal Republic is established. Each of the 32 states has a local constitution, but the Federal Constitution overrides lower sources of law at all times—and, from 2011, also international conventions related to human rights protections—and local constitutions must be aligned with the Federal Constitution's provisions.

The Supreme Court exercises constitutional control and plays an important role in interpreting the Federal Constitution.^34^ It has the power to strike down any local law that contradicts or contests the Federal Constitution, and it can also pronounce itself in favor of the constitutionality of any challenged law. There are two ways to create *jurisprudence*, which is analogous to *precedent/case law* in common law systems.^35^ The first is stipulated by the reiteration criteria. According to this criteria, rulings made by the Supreme Court and all federal judges and magistrates—which are called *relevant resolutions*—create *jurisprudence* when five relevant resolutions are ruled using the same line of legal reasoning. The second way to create *jurisprudence* is when eight of the eleven members of the Supreme Court agree on the main point of a particular relevant case. Jurisprudence of the Supreme Court is binding to all lower courts due to its hierarchy within the judicial system.

## Mexico's Rule of Law and Assisted Reproduction

The Federal Constitution neither defines a human embryo nor expressly defends human life from the moment of conception or fertilization. Despite this, conservatives have tried to argue that human life is protected from its beginning under Article 1 of the Federal Constitution^36^:

(…) Any discrimination based on ethnic or national origin, gender, age, disabilities, social status, health condition (…) or any other reason which attempts against human dignity and which is directed to either cancel or undermine people's rights and liberties is prohibited.^37^

According to conservatives, abortion and the destruction of human embryos are an affront to the right to life and the human dignity of those destroyed or terminated. Thus, these practices are legally prohibited. Although the Supreme Court recently favored this interpretation^38^ in a ruling regarding abortion due to severe congenital conditions, it established in its latest ruling (also regarding abortion) that:

It is clear that from a plain reading of the Mexican Constitution, we did not explicitly find in any of its text the institution of a specific right to life, the value of life, or other expression that allows to determine that life has a specific normative protection through a prohibition or mandate directed at the state's authorities.^39^

Diverging from previous rulings on abortion, where the focus was on civil law which protects the interests and rights of the unborn, in its latest ruling the Supreme Court favored women's reproductive rights over the life of the unborn and focused on the extent of protection of the constitutional right to life.^40^ In practical terms, this ruling means that there is no recognized right to life from the moment of conception or fertilization at the federal level, and thus *local abortion laws* that allow for on-demand abortion up to the 12th week (such as those of the State of Mexico City) do not contradict the Federal Constitution.^41^

Additionally, this ruling found that articles protecting human life that are in, or can be derived from, international declarations, covenants, and treaties of which Mexico is signatory do not expressly establish when life begins or the moment from which it should be protected. An exception to this finding is provided in Article 4 of the American Convention on Human Rights, of which Mexico is signatory. This article asserts that: ‘Every person has the right to have his life respected. This right shall be protected by law and, in general, from the moment of conception. No one shall be arbitrarily deprived of his life’.^42^ Although this article asserts that human life should, in general, be protected from the moment of conception, Mexico is not obliged to recognize this because the Mexican government made an interpretative declaration of that specific article at the moment of ratification. They considered the decision to protect life ‘in general’ from the moment of conception as one that should be taken by each individual state.

In relation to paragraph 1 of Article 4, [the Mexican government] considers that the expression ‘in general’ used in such paragraph does not constitute an obligation to adopt or maintain in force legislation that protects life ‘from the moment of conception’ since such matters belong to the reserved dominion of the States^43^

At the federal level, the General Health Law^44^ (a secondary piece of legislation) provides the legal definition of an embryo. Before commenting on this definition we will briefly elaborate on this law. The General Health Law regulates the right to the protection of health afforded to every person according to Article 4 of the Federal Constitution. In addition to the General Health Law, each state has its own health law. But, as described above, when there is conflict between a local health law and the General Health Law, the latter overrules the former.

It is reasonable to suppose that a legal definition of an embryo would appear in the section of the law dedicated to assisted reproduction. However, the General Health Law *does not specifically regulate assisted reproduction*.^45^ For at least 10 years, amendments to this law have been discussed in Congress, but, thus far, none have been successfully made into law. This important legal lacuna is recognized by academics, stakeholders, and politicians from all political parties, who have repeatedly called for legislation to be passed on this important subject. Although there are no specific assisted reproduction laws, the General Health Law does provide a definition of what should be understood, for legal purposes, as an embryo in the title: ‘Donation, Transplants and Loss of Life’. In Article 314, section VIII of this title, an embryo is defined as ‘the product of conception from the moment of it, and until the end of the twelfth gestational week’.^46^ Related to this, Article 330 section II states that the use of embryonic or foetal tissues that are the *product of induced abortions* is forbidden, regardless of the goal of such use. At this point, it should be clear that, *in relation to Article 330*, MRTs do not break the law, since they do not require tissues of this kind.

A consequence of the lack of specific regulation concerning assisted reproduction is that, at the federal level, no organization or authority regulates, evaluates, and compiles information about the way in which ARTs are carried out in Mexico, or the persons who carry them out.^47,48^ It also means, among other things, that there is no legal certainty about what kind of information should be collected for epidemiological and legal purposes, and the length of time that gametes and embryos should be stored. In terms of actual clinical practice, the Mexican Association of Reproductive Medicine and the Latin American Network of Assisted Reproduction (RedLara)—among other professional bodies—provide recommendations and regulations relating to the practice of assisted reproduction in Mexico. Nonetheless, clinics that offer assisted reproductive services follow their recommendations and regulations only on a voluntary basis.

At this point, it could be thought that, in terms of the General Health Law, there are no restrictions on MRTs in Mexico. But surprisingly, the *Regulations of the General Health Law on Health Research* include some directives that apply to MRTs. This regulatory document is an independent body of text (in the sense that it is not contained within the General Health Law) and, as its names suggests, further regulates certain aspects of the General Health Law. Article 56 of this regulation asserts that:

Research on assisted fertilization will only be admissible when it is applied to *solve sterility problems that cannot be solved otherwise* [emphasis added], respecting the couple's moral, cultural, and social point of view, even if these differ from those of the researcher.^49^

The text defines ‘assisted fertilization’ in a way that covers fertility practices carried out in vitro, including MRTs.^50^ Thus, if research on MRTs is used to solve sterility^51^ problems that cannot be otherwise solved, then it would not violate Article 56; if research on MRTs is *not used* to solve sterility problems that cannot be otherwise solved, then it *would* violate Article 56. It should be clear that, at this point in time, all applications of MRTs are experimental in nature^52^, and according to Article 3 section III of the Regulations of the General Health Law on Health Research they should be regarded as part of health research, since they entail actions that contribute to the prevention and control of health problems.^53^

Now, if a woman's eggs have mitochondria with deleterious DNA mutations such that the embryos produced with them will never be able to implant (making pregnancy impossible), then research on MRTs to solve this problem does not violate Article 56. This is because MRTs solve a sterility problem that cannot be otherwise solved. On the other hand, research on MRTs (ie research on assisted fertilization) for helping *fertile women* to have children without a mtDNA disease *would violate Article 56.* This is so because research on MRTs is not intended to solve sterility problems that cannot be solved otherwise.

At this point, we can conclude that—*assuming this interpretation of the law is correct and with the information we possess about the case*—Zhang's team violated Article 56 of this regulation.^54^ They did so because their research on MST included a woman *who could get pregnant* and deliver a live baby. In other words, research on MST was not used to solve a *sterility problem that could not be otherwise solved*. The Regulations of the General Health Law on Health Research do not establish a specific sanction for the violation of this article; however, they do assert that sanctions for violations of these regulations will be established by ‘the law’, in this case the General Health Law.

Article 416 of the General Health Law asserts that violations to this law and its regulations (eg Regulations of the General Health Law on Health Research) will incur administrative sanctions.^55^ In Article 417, these sanctions are outlined: (a) a warning with a subpoena, (b) a fine, (c) a temporal or definitive shutdown that can be partial or total, and (d) incarceration for up to 36 hours.^56^ The health authorities determine which of the above apply in each specific case. If the authorities decide on a fine, then its amount is established in conformity with Article 422, and can go up to 16,000 times the minimum wage (which roughly equates to 61,953.64 US dollars).^57^

Furthermore, another sanction of the General Health Law could apply to the violation of Article 56, depending on the fact if research on MRTs can be classified as research *on human beings*. It is true that carrying out MST does not equate to carrying out research on a human being; however, when we consider MST in conjunction with the woman who will be pregnant we realize that this research possesses a central human element, and thus could be constructed as research on a human being. Article 101 of the General Health Law dictates that persons carrying out research on human beings in contravention of this law or other applicable provisions (eg Regulations of the General Health Law on Health Research) are worthy of sanctions.^58^

Now, *if* research on MRTs is considered research on human beings, then *in this particular case* a violation of Article 56 of the Regulations of the General Health Law on Health Research would engage Article 101 of the General Health Law. The sanctions for a violation of Article 101 of the General Health Law are found in Article 421, which states that those who violate Article 101 will receive a fine between 6000 and 12,000 times the minimum wage (which roughly equates to a fine between 23,271.83 and 46,543.66 US dollars).^59^ These sanctions are meant to be applied by the Federal Commission for the Protection Against Sanitary Risks (COFEPRIS).^60^

In conclusion, *under our interpretation of the law and with the available information about the case*, we can assert that Zhang's team broke the Regulations of the General Health Law on Health Research, and that it is very probable that they also broke regulations regarding research on human beings. This conclusion, obviously, stands in stark contrast to Zhang's team's statements about the legality of their research in Mexico.

## Mexico and the Right to Life

**Figure 1.**
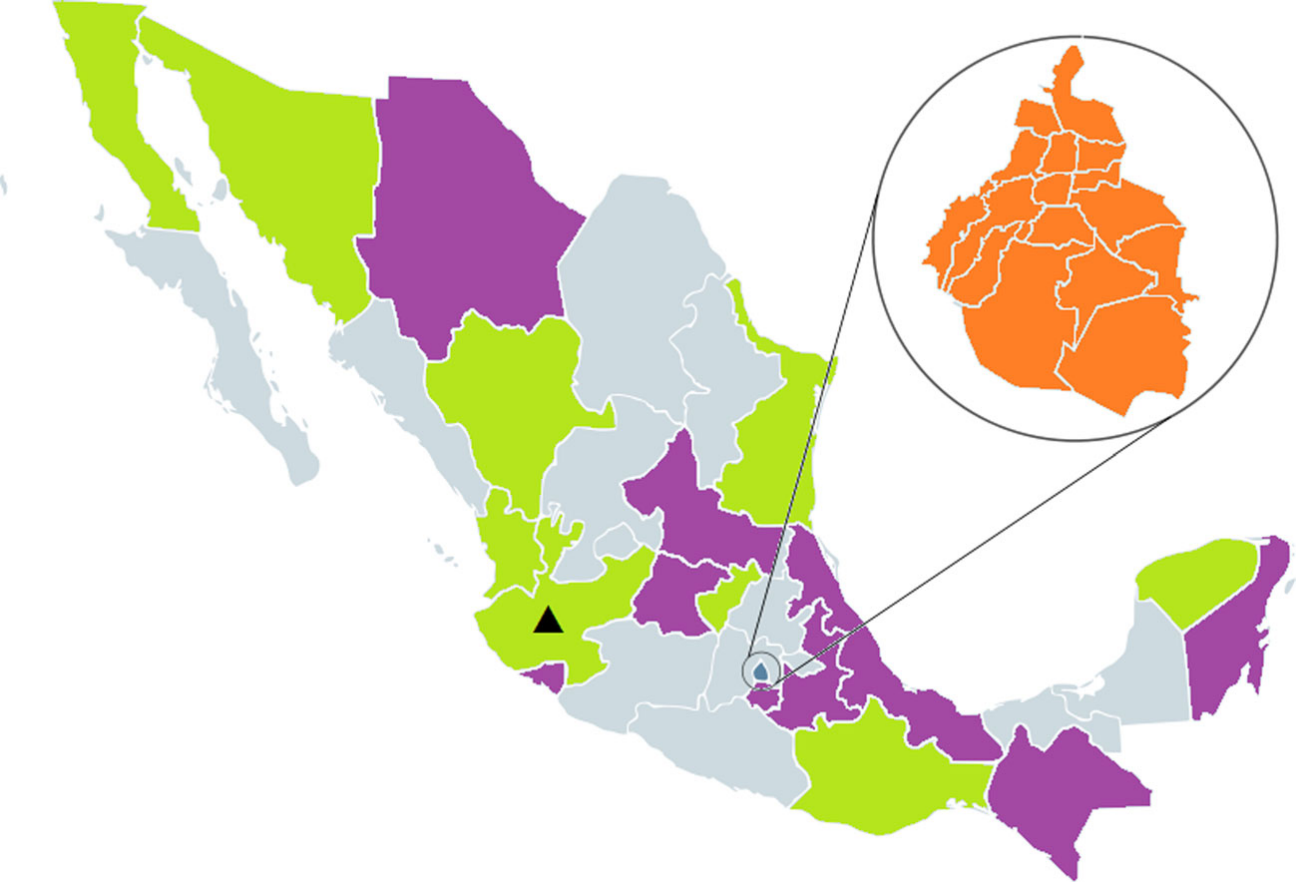
▴ State where MST was carried out. Purple, states where human life is protected from the moment of *conception* (ie implantation). Green, states where PNT is prohibited because human life is protected from the moment of *fertilization*. Orange, state where PNT is prohibited if the would-be-enucleated embryo is first created for a non-reproductive purpose.

Another way in which we can elucidate the instances in which MRTs and certain applications of MRTs are legally prohibited in Mexico, in addition to the specifications in Article 56, is to shift our attention from federal laws and regulations to state laws and regulations. Doing so allows us to identify states with laws that protect life from the moment of *fertilization*. We can then determine which states legally permit PNT, because PNT involves the intentional destruction of a single cell human embryo and this would be prohibited under such laws.^61^ At this point, let's remember two things: Mexico's Federal Constitution does not protect life from the point of conception or fertilization and laws protecting human life from the point of conception would not apply to PNT (given that conception is understood as the moment at which implantation occurs, and the destruction of human embryos for PNT occurs prior to implantation). In Mexico, 18 local constitutions protect life from the point of conception or fertilization (see Fig. 1).

Interestingly, Jalisco is one of the states that constitutionally protects life from the moment of fertilization. In this way, it clearly diverts from the Federal Constitution:

Article 4. (…) Likewise, the State of Jalisco recognizes, protects and underwrites the right to life of every human being, by expressively maintaining that from the moment of fertilization he is under the protection of the law and that he is reputed as born for all legal effects, until his natural death.^62^

Let's remember that Zhang's team carried out MST in the city of Guadalajara, which is within the state of Jalisco. This means two things. First, if Zhang's team *had carried out* PNT (which they did not), then they would have broken this law. Secondly, if after MST they destroyed the other three aneuploid embryos, while in Jalisco, then these actions would have clearly violated Article 4 of the state's constitution. At this point, we do not know the embryos’ fate. We do not know if they were destroyed, cryopreserved, or taken to another jurisdiction.

If Zhang's team were to declare that they intentionally destroyed the aneuploid embryos while in Guadalajara, or Jalisco, then we might question whether the state could try to prosecute them. Given that there is no precedent for this type of case (ie cases relating to MRTs), we must look to other cases in which persons have been prosecuted by the state for intentionally destroying embryos: abortion cases. Jalisco is among the most conservative states in Mexico and in addition to having laws that protect human life from the moment of fertilization it has a very punitive penal code in regard to abortion. Among Mexican states, Jalisco has one of the highest percentages of prosecution against women who abort, or who are suspected of having had an abortion.

Thirty-two women were prosecuted from 2007 to 2012 under such charges, and, of these, 25 were convicted.^63^ Despite this, a person who intentionally destroys a human embryo in vitro while carrying out ARTs, or as part of ART research (eg MRTs), cannot be prosecuted under abortion laws. This is because Jalisco's penal code, Article 227, defines abortion as ‘the death of the product of conception at any time during pregnancy’.^64^ It should be obvious that *without a pregnancy* there can be no abortion.

Another option for the state would be to prosecute the intentional destruction of embryos as a homicide.^65^ The state could follow this path because Article 213 of the state's penal code asserts that: ‘From twelve to eighteen years in prison will be imposed to the person that takes the life of another one’.^66^ And section I of Article 214 asserts that an injury will be considered fatal and thus sanctions of Article 213 will engage when: ‘the death is due to the alterations caused by the injury to the interested organ or organs, or that death is due as immediate or determinate consequence of the injury’.^67^ These articles could apply to cases of embryo destruction during PNT or after MST because, in Jalisco, a single cell embryo is, for all legal purposes, reputed as born from the moment of fertilization. Even if the embryos would die naturally if they were to be transferred to a woman, their *intentional destruction* could still be prosecuted since, in legal terms, their destruction would be akin to intentionally killing someone with a condition that will kill her in a couple of hours.

In conclusion, in Mexico, nine states prohibit PNT through laws protecting human life from the moment of fertilization. In Jalisco, specifically, the intentional destruction of human embryos could be prosecuted under criminal charges as homicide.

## Mexico and Genome Modification and Genetic Engineering

In Mexico, no federal law specifically concerns human genetic engineering or human genome modification. The General Health Law has a section on The Human Genome^68^, but it simply addresses the gathering and use of human genetic information. As a matter of fact, this section of the General Health Law does not engage with human genome modification or human genetic engineering of any kind.^69^

The only piece of federal legislation that directly addresses genetic engineering and genome modification is the 2005 Law on the Biosafety of Genetically Modified Organisms (GMOs). This piece of federal legislation was created with the intention of managing actual and possible risks related to the use and development of GMOs, in addition to promoting the development of this scientific area. Although this law regulates GMOs—and thus it is reasonable for us to expect to find in it regulations regarding human genetic engineering and human genome modification—it explicitly excludes humans from its oversight by means of its definition of GMOs. In Article 3, section XXI it asserts:

Genetically modified organism: Any living organism, *with the exception of human beings* [emphasis added], that has acquired a novel genetic combination, generated through the specific use of techniques of modern biotechnology (…)^70^

Thus, at the federal level, there is no law prohibiting human genetic engineering or genome modification at either the somatic or the germline level. Therefore, MRTs are not prohibited under this law. Although there is no federal law covering this ground, Mexico is signatory to international declarations that address the modification of the human germline. For example, Mexico is signatory of the Universal Declaration on the Human Genome and Human Rights, which explicitly addresses human germline modifications:

Article 24. (…) [The International Bioethics Committee of UNESCO] should make recommendations, in accordance with UNESCO’s statutory procedures, addressed to the General Conference and give advice concerning the follow-up of this Declaration, in particular regarding the identification of practices that could be contrary to human dignity, such as germ-line interventions.

Although this article states that germline interventions could be contrary to human dignity, the conditional form of this claim, ‘could be’, is of paramount importance. This means that in terms of the international covenants, treaties, and declarations that Mexico has signed and ratified, there is *no explicit prohibition* against PNT or MST that Mexico must follow.

At the state level the State of Mexico City (previously the Federal District) is the only state with laws regarding *human genetic manipulation*. Its penal code has an entire chapter (Chapter II) on genetic manipulation and the criminal sanctions that will be imposed on those who break such laws. Despite this, it is quite a short chapter, with only two articles. It is also interesting to note that the nature of the sanctions is criminal, whereas the General Health Law mainly imposes administrative sanctions. Article 154 of this penal code establishes that:

Between two and six years in prison, disqualification, suspension for the same amount of time from working in posts, jobs or public commissions, profession or trade, will be imposed to those who:

1. with different goals to the elimination or diminishment of grave diseases or maladies, manipulates human genes such that the genotype is altered;
2. fertilize human oocytes for any goal different to human procreation; and
3. create human beings by means of cloning or carry genetic engineering procedures for illicit ends.^71^

It is clear that carrying out MRTs in the State of Mexico City in order to help women or couples have children without an mtDNA disease does not violate section I of Article 154—taking into account that Article 56 of Regulations of the General Health Law on Health Research should be followed. In terms of section II of this article, creating an embryo with the intention of later on enucleating it is prohibited. This means that PNT is prohibited when, during its procedure, an embryo is created for a non-reproductive purpose, as is the case when an embryo is created with the sole intention of later enucleating it.

Interestingly, not all instances of PNT are prohibited under section II. PNT practices are legally permitted in the State of Mexico City if the would-be-enucleated embryos are first created for a reproductive purpose. For example, a couple going through ‘normal’ IVF could produce five embryos and end up with three surplus embryos if the first cycle results in a child. This couple could later decide to donate their three surplus embryos to a woman with an mtDNA disease who is considering PNT, supposing that the embryos have been cryopreserved at the right moment for PNT to be carried out. If doctors later enucleate these three embryos to carry out PNT, *they would not violate section II*, since the enucleated embryos were originally created for a reproductive purpose. Pointing out this caveat is important because we can now appreciate that in the State of Mexico City PNT *is not absolutely prohibited*. Additionally, the State of Mexico City does not protect life from the moment of conception or fertilization and does not explicitly prohibit the destruction of early embryos (see Fig. 1).

In conclusion, Mexico has no federal laws regulating human genome modification or human genetic engineering, and the international documents of which Mexico is signatory do not expressly prohibit human genome modification or human genetic engineering at the somatic or germline level. In the State of Mexico City, PNT is only prohibited when would-be-enucleated eggs are intentionally created for a non-reproductive purpose.

## Conclusion

Here we will briefly present our main conclusions and then explore Mexico's panorama of MRTs after this important event. First, in Mexico no federal laws specifically prohibit MRTs or regulate human genome modification or human genetic engineering. Secondly, at the federal level, research on MRTs is only legal when it is conducted to treat infertility that cannot otherwise be solved. Thus, *under our interpretation of the law and with the available information*, Zhang's team broke the Regulations of the General Health Law on Health Research and probably also regulations relating to research on human beings. Thirdly, PNT is prohibited in nine states due to laws protecting human life from the moment of fertilization. In the state of Jalisco, the intentional destruction of human embryos in vitro could be prosecuted under criminal charges as homicide, and in the State of Mexico City PNT is prohibited when a would-be-enucleated embryo is created for a non-reproductive end.

It is clear that Mexico needs laws at the federal and state level regulating assisted reproduction, in order to provide legal certainty about the obligations and rights of all the parties involved in it. It is also clear that laws concerning human genome modification and human genetic engineering are needed at both levels, in order to tackle the challenges that biotechnology offers. It could be thought that Zhang's team's actions might have prompted an informed public discussion among Mexican politicians, academics, stakeholders, and lay people regarding the paramount importance of moving toward scientifically sound and well-considered laws on both issues. However, the reality is otherwise. In order to understand what has happened in Mexico after Zhang's team's feat, we must take a step back.

Before the news broke on the first use of MST in a human reproductive setting, amendments relating to assisted reproduction to the General Health Law were being discussed in the Mexican Congress. The proposed amendments that seem more likely to pass are very restrictive (eg prohibiting surrogacy for same-sex couples or single persons), and scientifically problematic not only for assisted reproduction but also for other biological research areas (eg hybrid or chimera research).^72^ Academics, stakeholders, and NGOs had all raised concerns about these amendments and there is an ongoing public debate about them and the need for them to be rejected.^73^

Since Zhang's feat, *conservative politicians* have claimed that while the amendments they propose are ‘perfectible’ it is better at this time to enact them rather than to remain open to more experiments like Zhang's taking place.^74^ Thus, Zhang's team's actions have, unwittingly, helped the conservative cause make a stronger case for poorly considered legislation to be passed in a developing country.^75^ Further, and paradoxically, Zhang's team's actions have helped the case for passing legislation in Mexico that would legally prohibit MRTs, and which would certainly have economic effects on his fertility clinics in Mexico due to restrictions on who can access ARTs. In terms of research, for example, Article 71 bis 6 of the proposed amendments asserts that the creation of genetically modified embryos is prohibited; experimentation with or on embryos is prohibited; creation of embryos for non-reproductive ends is prohibited; transportation of sperm, oocytes or embryos out of the country is prohibited; and any kind of selection against disability is prohibited.^76^ It is clear that while the birth of the first post-MST baby has prompted a discussion of MRTs in Mexico, it has also helped those who wish to bar MRTs and related practices from happening in the country.

## Footnotes

1 Jessica Hamzelou, *World's First Baby Born With New ‘3 Parent’ Technique New Scientist* (2016), https://www.newscientist.com/article/2107219-exclusive-worlds-first-baby-born-with-new-3-parent-technique/ (accessed Oct. 3, 2016).

2 Leigh's syndrome is a devastating neurological disorder. It is characterized by the degeneration of the central nervous system, which causes psychomotor regression and loss of mental abilities. Most patients die a few years after its diagnosis.

3 John Zhang et al., *First Live Birth Using Human Oocytes Reconstituted by Spindle Nuclear Transfer for Mitochondrial DNA Mutation Causing Leigh Syndrome*, 106 Fertil. Steril. e375–e376 (2016); Kate Johnson, *First Spindle Nuclear Transfer Baby Has Low Mutant DNA Load Medscape* (2016), http://www.medscape.com/viewarticle/870766 (accessed Dec. 7, 2016); *Judge Science on Merit, Not Assumptions*, 539 Nature News 139 (2016); Medscape, *Dr John Zhang Speaking At #artworldcongress About Baby Born With DNA from 3 People* (2016), https://www.periscope.tv/Medscape/1BRJjANLwVgGw# (accessed Dec. 7, 2016).

4 James Gallagher, *UK Approves Three-Person Babies*, BBC News, Feb. 24, 2015, http://www.bbc.co.uk/news/health-31594856 (accessed Oct. 3, 2016).

5
Human Fertilisation and Embryology Authority, The Human Fertilisation and Embryology (Mitochondrial Donation) Regulations 2015 (2015), http://www.legislation.gov.uk/uksi/2015/572/pdfs/uksi_20150572_en.pdf (accessed Oct. 5, 2016).

6
Institute of Medicine of the National Academies, Mitochondrial Replacement Techniques: Ethical, Social, and Policy Considerations (2016), http://www.nap.edu/21871 (accessed Mar. 3, 2016).

7 Charles Dent, Text - H.R.2029 - 114th Congress (2015–2016): Consolidated Appropriations Act, 2016 Sec. 749 (2015), https://www.congress.gov/bill/114th-congress/house-bill/2029/text (accessed Oct. 3, 2016).

8
Zachary Brennan, Expert Committee: FDA Should Allow Mitochondrial Replacement Trials Under Certain Conditions, Regulatory Affairs Professionals Society (2016), http://raps.org/Regulatory-Focus/News/2016/02/03/24245/Expert-Committee-FDA-Should-Allow-Mitochondrial-Replacement-Trials-Under-Certain-Conditions/ (accessed Oct. 4, 2016).

9 I. Glenn Cohen & Eli Y. Adashi, *Preventing Mitochondrial DNA Diseases: One Step Forward, Two Steps Back*, 316 JAMA 273–274 (2016).

10 Bob Livingston, H.R.2880, 104th Congress (1995–1996): Balanced Budget Downpayment Act, I Sec. 128 (1996), https://www.congress.gov/bill/104th-congress/house-bill/02880 (accessed Oct. 3, 2016).

11 In cytoplasmic transfer, cytoplasm from a donor egg is transferred into a second egg to make up for a presumptive ‘ooplasmic deficiency’. This technique was indented to help women who had experienced repeated poor embryo development and implantation failure. Jason A. Barritt et al., *Cytoplasmic Transfer in Assisted Reproduction*, 7 Hum. Reprod. Update 428–435 (2001); Martin H. Johnson, *Setting the Record Straight*, 33 Reprod. BioMed. Online 657–658 (2016).

12 Charlotte Pritchard, *The Girl With Three Biological Parents*, BBC News, Sept. 1, 2004, http://www.bbc.co.uk/news/magazine-28986843 (accessed Oct. 14, 2016).

13 Hamzelou, *supra* note 1.

14 Two things bear noticing. First, many media headlines have claimed that the baby born after MST was the first baby to have been intentionally produced with genetic material from three persons. Though this makes for a catchy headline, children were produced with genetic material from three persons in the late 1990s after ooplasmic transfer. C. A. Brenner et al., *Mitochondrial DNA Heteroplasmy After Human Ooplasmic Transplantation*, 74 Fertil. Steril. 573–578 (2000). Second, and most important, after this paper was accepted for publication Alejandro Chavez-Badiola, the director of New Hope Fertility Center in Mexico, announced that they are planning to use MST at least 20 times in the first half of 2017. Michael Le Page, *Mexico Clinic Plans 20 ‘Three-Parent’ Babies in 2017 New Scientist* (2016), https://www.newscientist.com/article/2115731-exclusive-mexico-clinic-plans-20-three-parent-babies-in-2017/ (accessed Dec. 12, 2016).

15 Although it is an important topic, in this paper we will not discuss the legal status of surrogacy in Mexico or its regulation through different state level civil codes—statutory law—in places such as Tabasco and Sinaloa.

16 Mexican law does not differentiate between ‘conception’ and ‘fertilization’ and most of the times these concepts are used interchangeably. Even when this is the case, in a 2012 ruling—Artavia Murillo et al. (‘In Vitro Fertilization’) v. Costa Rica—the Inter-American Court of Human Rights clarified what should be understood for conception: ‘the [Inter-American] Court [of Human Rights] understands the word “conception” from the moment at which implantation occurs’ Inter-American Court of Human Rights, Case of Artavia Murillo et al. (‘In vitro fertilization’) v. Costa Rica, 57 (2012), http://www.corteidh.or.cr/docs/casos/articulos/seriec_257_ing.pdf (accessed Jun. 12, 2016). In this paper, we follow the Inter-American Court on Human Rights’ position regarding ‘conception’. We do this because in Mexican law the jurisprudences of the Inter-American Court of Human Rights are binding—when Mexico is not part of the judgment—when they give more protections to the person than the Mexican law. Suprema Corte de Justicia de la Nación, Sesión Pública Núm. 89 Ordinaria (2013), https://www.scjn.gob.mx/sites/default/files/actas-sesiones-publicas/documento/2016-11-15/89%20-%203%20DE%20SEPTIEMBRE%20DE%202013_0.pdf (accessed Oct. 4, 2016). For an analysis of how the jurisprudences of the Inter-American Court of Human Rights have, historically, interacted with the Mexican law, see Sandra Carrizosa-Guzmán, *Alcance de la Jurisprudencia de la Corte Interamericana de Derechos Humanos en México: Reflexiones en torno a la protección de la Salud, in*La norma constitucional mexicana, paradigma de transformación social. Obra en homenaje a los cuarenta años de vida académica del Doctor Mariano Palacios Alcocer 71–96 (Mariana Leoba Castañeda Rivas, et al. ed., 2014).

17 On identity issues, see Françoise Baylis, *The Ethics of Creating Children with Three Genetic Parents*, 26 Reprod. Biomed. Online 531–534 (2013); Anthony Wrigley, Stephen Wilkinson & John B. Appleby, *Mitochondrial Replacement: Ethics and Identity*, 29 Bioethics 631–638 (2015); S. Matthew Liao, *Do Mitochondrial Replacement Techniques Affect Qualitative or Numerical Identity?*, 31 Bioethics 20–26 (2017). On transgenerational health risks, see John B. Appleby, *The Ethical Challenges of the Clinical Introduction of Mitochondrial Replacement Techniques*, 18 Med. Health Care & Philos. 501–514 (2015); Baylis, *id.* Annelien L. Bredenoord et al., *Avoiding Transgenerational Risks of Mitochondrial DNA Disorders: A Morally Acceptable Reason for Sex Selection?*, 25 Hum. Reprod. 1354–1360 (2010); Martin H. Johnson, *Tri-parenthood—A Simply Misleading Term or an Ethically Misguided Approach?*, 26 Reprod. BioMed. Online 516–519 (2013). On the disclosure of MRT conception, see John B. Appleby, *Should Mitochondrial Donation Be Anonymous?*, J. Med. & Philos. (2016); Appleby (2015), ibid. Reuven Brandt, *Mitochondrial Donation and “The Right to Know,”* 42 J. Med. 678–684 (2016); Inmaculada de Melo-Martin, *When the Milk of Human Kindness Becomes a Luxury (and Untested) Good. A Reply to Harris’ Unconditional Embrace of Mitochondrial Replacement Techniques*, Camb. Q. Healthcare Ethics (2017); John Harris, *How To Welcome New Technologies: Some Comments on Inmaculada De Melo-Martin*, Camb. Q. Healthcare Ethics (2017); John Harris, *Germline Modification and the Burden of Human Existence*, 25 Camb. Q. Healthcare Ethics 6–18 (2016); John Harris, *Germline Manipulation and Our Future Worlds*, 15 Am. J. Bioethics 30–34 (2015); César Palacios-González, *Resource Allocation, Treatment, Disclosure, and Mitochondrial Replacement Techniques: Some Comments on de Melo-Martin and Harris*, Camb. Q. Healthcare Ethics (2017); Monika W. Piotrowska, *Why is an Egg Donor a Genetic Parent, But Not a Mitochondrial Donor?*, Camb. Q. Healthcare Ethics (2017). On genealogical ancestry, see Baylis, *supra* note 17; César Palacios-González, *Mitochondrial Replacement Techniques: Egg Donation, Genealogy and Eugenics*, 34 Monash Bioethics Rev. 37–51 (2016). On first in-human use, see Annelien L. Bredenoord & Peter Braude, *Ethics of Mitochondrial Gene Replacement: From Bench to Bedside*, 341 BMJ c6021 (2010); Institute of Medicine of the National Academies, *supra* note 6; Nuffield Council on Bioethics, Novel techniques for the prevention of mitochondrial DNA disorders: an ethical review (2012). On the role of the egg donor, see Erica Haimes & Ken Taylor, *Rendered Invisible? The Absent Presence of Egg Providers in U.K. Debates on the Acceptability of Research and Therapy for Mitochondrial Disease*, 33 Monash Bioethics Rev. 360–378 (2015). On the value of MRTs, see Tina Rulli, *What Is the Value of Three-Parent IVF?*, 46 Hastings Cent. Rep. 38-47 (2016).

18 See Sarah Chan & Maria de Jesús Medina-Arellano, *Genome Editing and International Regulatory Challenges: Lessons From Mexico*, 2 Ethics Med. & Pub. Health 426–434 (2016); I. Glenn Cohen, Julian Savulescu & Eli Y. Adashi, *Transatlantic Lessons in Regulation of Mitochondrial Replacement Therapy*, 348 Science 178–180 (2015); Tetsuya Ishii, *Potential Impact of Human Mitochondrial Replacement on Global Policy Regarding Germline Gene Modification*, 29 Reprod. Biomed. Online 150–155 (2014); Guido Pennings, *International Harmonization and Mitochondrial Replacement*, 29 Reprod. Biomed. Online 269 (2014); Johanna Schandera & Tim K. Mackey, *Mitochondrial Replacement Techniques: Divergence in Global Policy*, 32 Trends Genet. 385–390 (2016).

19 This section draws from César Palacios-González, *Ethics of Mitochondrial Replacement Techniques: A Habermasian Perspective*, 31 Bioethics 27–36 (2017).

20 This could change if functional oocytes were to be generated from male cells. Henry T. Greely, The End of Sex and the Future of Human Reproduction (1st ed., 2016); César Palacios-González, John Harris & Giuseppe Testa, *Multiplex Parenting: IVG and the Generations to Come*, 40 J. Med. Ethics 752–758 (2014).

21 Stephen Anderson et al., *Sequence and Organization of the Human Mitochondrial Genome*, 290 Nature 457–465 (1981); Robert W. Taylor et al., *The Determination of Complete Human Mitochondrial DNA Sequences in Single Cells: Implications for the Study of Somatic Mitochondrial DNA Point Mutations*, 29 Nucleic Acids Res. e74–e74 (2001).

22
Nuffield Council on Bioethics, *supra* note 17, at vii.

23 ‘During the production of primary oocytes, a selected number of mitochondrial DNA (mtDNA) molecules are transferred into each oocyte. Oocyte maturation is associated with the rapid replication of this mtDNA population. This restriction-amplification event can lead to a random shift of mtDNA mutational load between generations and is responsible for the variable levels of mutated mtDNA observed in affected offspring from mothers with pathogenic mtDNA mutations’ Robert W. Taylor & Doug M. Turnbull, *Mitochondrial DNA Mutations in Human Disease*, 6 Nat. Rev. Genet. 389–402, 393 (2005).

24
UK Department of Health, Mitochondrial donation: A consultation on draft regulations to permit the use of new treatment techniques to prevent the transmission of a serious mitochondrial disease from mother to child (2014), https://www.gov.uk/government/uploads/system/uploads/attachment_data/file/285251/mitochondrial_donation_consultation_document_24_02_14_Accessible_V0.4.pdf (accessed Sept. 14, 2015).

25 Andrew M. Schaefer et al., *Prevalence of Mitochondrial DNA Disease in Adults*, 63 Ann. Neurol. 35–39 (2008).

26 Hubert J. M. Smeets et al., *Preventing the Transmission of Mitochondrial DNA Disorders Using Prenatal or Preimplantation Genetic Diagnosis*, 1350 Ann. NY Acad. Sci. 29–36 (2015).

27 For a discussion on the appropriateness of this terminology, see Vardit Ravitsky, Stanislav Birko & Raphaelle Dupras-Leduc, *The ‘Three-Parent Baby’: A Case Study of How Language Frames the Ethical Debate Regarding an Emerging Technology*, 15 Am. J. Bioethics 57–60 (2015); Palacios-González (2016), *supra* note 17; Ainsley J. Newson & Anthony Wrigley, *Is Mitochondrial Donation Germ-Line Gene Therapy? Classifications and Ethical Implications*, 31 Bioethics 55–67 (2017); Françoise Baylis, *Human Nuclear Genome Transfer (So-Called Mitochondrial Replacement): Clearing the Underbrush*, 31 Bioethics 7–19 (2017).

28 Lyndsey Craven et al., *Pronuclear Transfer in Human Embryos to Prevent Transmission of Mitochondrial DNA Disease*, 465 Nature 82–85 (2010); Masahito Tachibana et al., *Mitochondrial Gene Replacement in Primate Offspring and Embryonic Stem Cells*, 461 Nature 367–372 (2009); Akiko Yabuuchi et al., *Prevention of Mitochondrial Disease Inheritance by Assisted Reproductive Technologies: Prospects and Challenges*, 1820 Biochim. Biophys. Acta 637–642 (2012).

29 In this paper, we will not address the philosophical question if the egg donor is, or not, a second genetic mother.

30 Craven et al., *supra* note 28; Louise A. Hyslop et al., *Towards Clinical Application of Pronuclear Transfer to Prevent Mitochondrial DNA Disease*, 534 Nature 383–386 (2016); Nuffield Council on Bioethics, *supra* note 17.

31
Nuffield Council on Bioethics, *supra* note 17; Tachibana et al., *supra* note 28.

32 Hyslop et al., *supra* note 30; Mitsutoshi Yamada et al., *Genetic Drift Can Compromise Mitochondrial Replacement by Nuclear Transfer in Human Oocytes*, 18 Cell Stem Cell 749–754 (2016); Eunju Kang et al., *Mitochondrial Replacement in Human Oocytes Carrying Pathogenic Mitochondrial DNA Mutations*, Nature (2016), http://www.nature.com/nature/journal/vaop/ncurrent/full/nature20592.html (accessed Dec. 7, 2016).

33 Until last year the political composition of the country was 31 states plus a federal district. The federal district is now the State of Mexico City.

34 For an account of the Supreme Court's recent history, see Alejandro Madrazo & Estefanía Vela, *The Mexican Supreme Court's (Sexual) Revolution?*, 89 Tex. L. Rev. 1863–1893 (2011).

35
Cámara de Diputados del H. Congreso de la Unión, Ley de Amparo, Reglamentaria de los Artículos 103 y 107 de la Constitución Política de los Estados Unidos Mexicanos Title four (2016), http://www.diputados.gob.mx/LeyesBiblio/pdf/LAmp_170616.pdf (accessed Oct. 14, 2016).

36 All translations here are our own unless otherwise indicated.

37
Congreso Constituyente, Constitución Política de los Estados Unidos Mexicanos (1917 as amended to Jan. 27, 2016), http://www.diputados.gob.mx/LeyesBiblio/htm/1.htm (accessed Oct. 14, 2016).

38
Suprema Corte de Justicia de la Nación, Tesis Jurisprudencial num. P./J. 14/2002 de Suprema Corte de Justicia, Pleno (Acciones de Inconstitucionalidad) (2002), http://suprema-corte.vlex.com.mx/vid/jurisprudencial-pleno-jurisprudencia-27193739 (accessed Oct. 14, 2016).

39
Suprema Corte de Justicia de la Nación, Acción de Inconstitucionalidad 146/2007 y su Acumulada 147/2007 153 (2008), https://www.unifr.ch/ddp1/derechopenal/temas/t_20090316_03.pdf (accessed Oct. 14, 2016).

40 Maria de Jesús Medina-Arellano, *Stem Cell Regulation in Mexico: Current Debates and Future Challenges*, 5 Stud. Ethics L. & Tech. 1–33, 17 (2011).

41 For a comment on this sentence, see Maria de Jesús Medina-Arellano, *The Need for Balancing the Reproductive Rights of Women and the Unborn in the Mexican Courtroom*, 18 Med. L. Rev 427–433 (2010).

42
Organization of American States, American Convention on Human Rights ‘Pact of San Jose, Costa Rica’ (1978), http://www.oas.org/dil/treaties_B-32_American_Convention_on_Human_Rights.htm (accessed Oct. 14, 2016). See note 16, for an account of the differences between ‘conception’ and ‘fertilization’ in Mexican Law.

43
Suprema Corte de Justicia de la Nación, *supra* note 39, at 173.

44 That for all intends and purposes is a national health law.

45 At present time, there are various assisted reproduction amendments to the General Health Law that are being discussed. Sara Reardon, *Mexico Proposal to Ban Human-Embryo Research Would Stifle Science*, 540 Nat. News 180 (2016); César Palacios-González, Una propuesta de ley que prohíbe embarazos y trasplantes Animal Político (2016), http://www.animalpolitico.com/blogueros-una-vida-examinada-reflexiones-bioeticas/2016/12/14/una-propuesta-ley-prohibe-embarazos-trasplantes/ (accessed Dec. 15, 2016).

46
Cámara de Diputados del H. Congreso de la Unión, Ley General de Salud (1984 as amended to Jan. 1, 2016), http://www.diputados.gob.mx/LeyesBiblio/pdf/142_010616.pdf (accessed Dec. 15, 2016).

47 At the state level, certain states have surrogacy laws but these laws do not refer to assisted reproduction per se, but to the rights and obligations of each party when entering into a surrogacy agreement.

48 It must be said that the Federal Commission for the Protection Against Sanitary Risks (COFEPRIS) establishes regulations for clinics offering assisted reproduction, but *only in terms of facilities*.

49
Cámara de Diputados del H. Congreso de la Unión, Reglamento de la Ley General de Salud en Materia de Investigación para la Salud (1987), http://www.diputados.gob.mx/LeyesBiblio/regley/Reg_LGS_MIS.pdf (accessed Dec. 15, 2016).

50 Article 40 section XI defines assisted fertilization as follows: ‘That in which the insemination is artificial (homologous or heterologous) and includes in vitro fertilization’. *Id.* Now, in vitro fertilization is not defined in this law. However, it is reasonable to contend that reproductive practices that inherently include in vitro fertilization fall under the umbrella of assisted fertilization.

51 Sterility is defined, elsewhere, as the failure to achieve a clinical pregnancy after 12 months of regular unprotected sexual intercourse. Instituto Mexicano del Seguro Social, Diagnóstico de la pareja infértil y tratamiento con técnicas de baja complejidad (2012), http://www.imss.gob.mx/sites/all/statics/guiasclinicas/621GRR.pdf (accessed Dec. 15, 2016).

52 If carrying out MRTs were common clinical practice, then Article 56 would not engage, as it is the case with the current practice of PGD, for example.

53 Article 3: ‘Health research entails carrying out actions that contribute: (…) III. To the prevention and control of health problems’ Cámara de Diputados del H. Congreso de la Unión, *supra* note 49.

54 Someone could claim that Zhang's team's actions were a research step toward solving sterility problems that cannot be solved otherwise, and thus legal. Even when this might seem compelling, we contend that there is a strong reason to doubt that Zhang's team's treatment of the Jordanian couple was only a step in research toward solving mtDNA diseases that cause sterility problems that cannot be solved otherwise. This is that when the team was, repeatedly, asked about Mexican regulations they never made this important precision, even when a big part of the whole controversy has revolved around MRTs regulations.

55
Cámara de Diputados del H. Congreso de la Unión, *supra* note 46.

56
*Id.*

57 Currency exchange rate calculated at 1 US dollar to 18.83 Mexican pesos (Oct. 10, 2016).

58
Cámara de Diputados del H. Congreso de la Unión, *supra* note 46.

59
*Id.*

60 María de Jesús Medina-Arellano, *The Rise of Stem Cell Therapies in Mexico: Inadequate Regulation or Unsuccessful Oversight?*, 2 Revista Redbioética/UNESCO 63–78 (2012).

61 In these states, the destruction of surplus embryos after both MRTs would also be legally prohibited.

62
Congreso Constituyente del Estado Libre y Soberano de Jalisco, Constitución Política Del Estado De Jalisco (1917 as amended to Apr. 10, 2014), http://www.jalisco.gob.mx/sites/default/files/constituci_n_politica_del_estado_de_jalisco.pdf (accessed Dec. 15, 2016).

63
Ximena Andión & Rebeca Ramos, Omisión e Indiferencia. Derechos Reproductivos en México 32 (2013), http://informe.gire.org.mx/caps/cap1.pdf (accessed Oct. 20, 2016).

64
Congreso del Estado de Jalisco, Código Penal Para el Estado Libre y Soberano de Jalisco (1982 as amended to Apr. 9, 2013), http://www.jalisco.gob.mx/sites/default/files/C%25C3%25B3digo%2520Penal%2520para%2520el%2520Estado%2520Libre%2520y%2520Soberano%2520de%2520Jalisco%2520%252826OCTU12%2529.pdf (accessed Oct. 15, 2016).

65 In Mexico, the concept ‘homicide’ is used to denote to what in the USA is referred to as ‘manslaughter’, whereas ‘typified homicide’ is used to refer to what in the USA is referred as ‘homicide’.

66
Congreso del Estado de Jalisco, *supra* note 64.

67
*Id.*

68
Cámara de Diputados del H. Congreso de la Unión, *supra* note 46 at Chapter Five Bis.

69 Chan & Medina-Arellano, *supra* note 18.

70
Cámara de Diputados del H. Congreso de la Unión, Ley de Bioseguridad de Organismos Genéticamente Modificados (2005), http://www.diputados.gob.mx/LeyesBiblio/pdf/LBOGM.pdf (accessed Oct. 15, 2016).

71
Asamblea Legislativa del Distrito Federal, Código Penal Para el Distrito Federal (2002 as amended to Sep. 10, 2013), http://www.fimevic.df.gob.mx/documentos/transparencia/codigo_local/CPDF.pdf (accessed Oct. 15, 2016).

72
Sylvana Beltrones Sánchez, Iniciativa que Reforma y Adiciona Diversas Disposiciones de la Ley General de Salud, a cargo de la diputada Sylvana Beltrones Sánchez, del Grupo Parlamentario del PRI (2016), http://gaceta.diputados.gob.mx/Black/Gaceta/Anteriores/63/2016/mar/20160301-IV/Iniciativa-12.html (accessed Oct. 15, 2016); Reardon, *supra* note 45; Palacios-González, *supra* note 45.

73 Héctor A. Mendoza C., *10 Argumentos Sobre la Ignorancia Legislativa*, Animal Político, Oct. 12, 2016, http://www.animalpolitico.com/blogueros-una-vida-examinada-reflexiones-bioeticas/2016/10/12/10-argumentos-la-ignorancia-legislativa/ (accessed Oct. 26, 2016); Katia D’Artigues Beauregard, *Maternidad Discriminatoria Gracias Presidenciales*, El Universal, Oct. 12, 2016, http://www.eluniversal.com.mx/entrada-de-opinion/columna/katia-dartigues/nacion/2016/10/12/maternidad-discriminatoria-gracias (accessed Oct. 17, 2016); Redacción Animal Político, *El Congreso Busca Restringir Acceso a Parejas a la Reproducción Asistida*, Animal Político, Sept. 23, 2016, http://www.animalpolitico.com/2016/09/ley-reproduccion-asistida-gestacion-subrogada/ (accessed Oct. 15, 2016); Reardon, *supra* note 45.

74 Our position is, although we will not defend it here since we are only interested in presenting the Mexican law as it currently stands, that all current proposed amendments to the General Health law should be rejected. In order to draft scientifically informed amendments to the General Health Law regarding ARTs Mexican politicians should include academics from the biological and medical sciences, ethicists, stakeholders and lay people in the legislative process. We also think that, given the actual scientific evidence and ethical arguments, MRTs should be allowed under a licensing scheme akin to that of the UK. One of us have defended the moral permissibility of MRTs elsewhere: Palacios-González (2016 and 2017), *supra* note 17; Palacios-González (2017), *supra* note 19.

75 Juan Paullier, ¿Por qué la concepción del bebé de ‘tres padres’ se realizó en México? BBC Mundo (2016), http://www.bbc.com/mundo/noticias-37491942 (accessed Oct. 16, 2016).

76
Beltrones Sánchez, *supra* note 72.

